# Dynamic mechanical characteristics and failure mode of serpentine under a three-dimensional high static load and frequent dynamic disturbance

**DOI:** 10.1371/journal.pone.0222684

**Published:** 2019-09-23

**Authors:** Chun Wang, Lu-ping Cheng, Cheng Wang, Zhu-qiang Xiong, Shi-ming Wei

**Affiliations:** 1 School of Energy Science and Engineering, Henan Polytechnic University, Jiaozuo Henan, China; 2 State and Local Joint Engineering Laboratory for Gas Drainage & Ground Control of Deep Mines, Henan Polytechnic University, Jiaozuo, Henan, China; 3 School of Resources and Safety Engineering, Central South University, Changsha, Hunan, China; 4 The Collaborative Innovation Center of Coal Safety Production of Henan, Jiaozuo, Henan, China; China University of Mining and Technology, CHINA

## Abstract

The improved split Hopkinson pressure bar (SHPB) was used to study the dynamic mechanical properties and failure characteristics of surrounding rock in deep rock mass engineering that is under high stress and affected by blasting excavation and other dynamic disturbances. In a three-dimensional high static load and frequent dynamic disturbance test, the preload high axial pressure and confining pressure are used to simulate the high crustal stress of deep rock, and the effect of small disturbances on the rock is simulated by the low impact load. The results show that there are two types of dynamic stress-strain curves of deep rock: an elastic-plastic curve and plastic-elastic-plastic curve. The curves consists of five parts: the compaction stage, micro-crack steady development stage, micro-crack unstable propagation stage, fatigue damage stage, and fatigue failure stage. Reductive phenomena of constringent strain after dynamic peak stress appear because of the different degrees of rock damage. Moreover, these phenomena include two conditions, namely, whether rebound occurs or not. The impact resistance of rock is strongest when the ratio of the confining pressure to axial pressure is optimal, and the dynamic average strength of rock and accumulative impact times decrease with the increase of the preloaded axial compression and increase with the increase of the preloaded confining pressure. Both the dynamic deformation modulus and dynamic peak stress decrease with the increase of the accumulative impact time, while the maximum strain and the dynamic peak strain increase. The corresponding rebound strain as a whole first increases and then decreases with the increasing impact times. For deep rock, tensile failure and single-bevel plane shear failure are the main failure modes, and pull-compression mixed friction failure is the auxiliary failure mode. Additionally, the lumpiness of broken rock decreases with the increase of the preloaded axial compression and increases with the increase of the preloaded confining pressure.

## Introduction

The demand for mineral resources is increasing gradually with the rapid development of the world economy and industrial technology. The exploitation of deep resources is unavoidable because mineral resources are non-renewable, especially metal mineral resources. However, the mechanical properties and failure characteristics of deep rocks are different from those of shallow rocks because deep rocks exist in the complex environment of high earth temperature, high karst water pressure, and high crustal stress, and they are also affected by blasting excavation and other dynamic disturbances [1–3]. Right now, the research on dynamic characteristics and failure characteristics of deep rocks is still in the exploratory stage. The law of deformation and failure of deep rocks in complex mechanical environments has not been fully mastered. Therefore, insufficient theoretical basis exists for the design and construction of deep rock mass engineering, which often causes disasters and accidents, such as rock burst, large-scale collapse and sloughing [4–5]. Thus, it very important to study the mechanical properties and failure characteristics of deep rocks in the corresponding mechanical environment based on deep rock mass engineering.

In recent years, the problem of dynamic mechanical properties of rocks in the deep complex mechanical environment has attracted the attention of experts in the field of rock mechanics. A large number of experimental studies have been carried out, and the results can lay the foundation for the study of dynamic properties and failure characteristics of deep rocks. To research the dynamic impact strength of deep rock in different complex mechanical environments, the improved split Hopkinson pressure bar (SHPB) has been used by scholars to carry out the dynamic impact disturbance test. The dynamic triaxial compressive strength of rocks under low confining pressures increases with the increase of strain rate and increases linearly with the increase of confining pressure when the only influencing factors considered are high strain rate and low confining pressure [6–8]. The dynamic mechanical properties and failure mechanism of rocks after high-temperature treatment were studied by a number of experts in the field of rock mechanics, with the only influencing factor considered being the high ground temperature environment. It was found that the dynamic impact strength of rocks decreases with the increase of temperature, and the higher the temperature is, the greater the degree of rock fragmentation is when the rocks are under the same impact load [9–15]. In deep mineral mining, the deep rocks are in a complex environment of high crustal stress, and they are also affected by blasting excavation. Based on this, the dynamic characteristics of rock under combined static and dynamic loading were researched by scholars. The results of one-static and dynamic combined loading tests show that the dynamic impact strength of rocks increases first and then decreases with the increase of the preloading axial pressure. When the preloaded axial pressure is approximately 60% the strength of rock under static load, the resistance of impact strength achieves the maximum value under the common action of axial static load and impact load. At the same time, the internal energy of rock experiences three stages (absorption-release-absorption). As a consequence, the failure mode of rock is ultimately the destruction of compression-shear [16–19]. The results of three-static and dynamic combined loading tests show that the dynamic impact strength of rocks and the deformation modulus of rocks were increased with the increases of confining pressure. Moreover, rocks have obvious characteristics that transform frangibility to ductility [20–23]. A primary study regarding the dynamic behavior of rock during its post-failure stage has been performed based on a scenario in which the surrounding rock of deep rock mass engineering is in the state of post-peak fracture and affected by blasting disturbance. It is found that the post-peak cracked rock experiences shear failure when subjected to the coupled static and dynamic loads, and the original crack influences the crack propagation direction during the process of impact failure [24–27]. Z.Q. Yin et al. [28–29] also explored the static and dynamic mechanical properties of deep coal and rock under different gas pressures. The results showed that the increase of gas pressure leads to the decrease of the uniaxial compressive strength of coal and rock. Based on the above introduction, a large number of experimental studies on the mechanical characteristics of deep rock have been carried out by scholars in the field of rock mechanics, which is combined with the actual mechanical environment of deep rock mass engineering. However, there are few experimental research studies concerning the deformation and failure characteristics of deep rock under frequent blasting excavation disturbance.

The surrounding rock is affected by frequent blasting excavation disturbance in deep rock engineering, especially in the mining process of deep mineral resources. Therefore, the three-dimensional high static load and frequent dynamic disturbance test was carried out, and its engineering background is the Dongguashan Copper Mine. The Dongguashan Copper Mine is located in Tongling City, Anhui Province, China. Because the extractive depth of the Dongguashan Copper Mine has already reached one kilometer, the deep surrounding rock of extracted ore drift produces an intricately destructive phenomenon under intensively high static stress and blasting disturbance [30–33]. In the three-dimensional high static load and frequent dynamic disturbance test, preload high axial pressure and confining pressure are used to simulate the high crustal stress of deep rock, and the effect of small disturbances on the rock is simulated by the low impact load. The research results can provide the theoretical basis for the support of surrounding rock in deep rock mass engineering, and it is of great significance to practical engineering.

## Test system and scheme

### Specimen preparation

The serpentine specimens used in the test were drilled from the wall rock of extracted ore drift in the Dongguashan copper mine, 900 m underground. The Dngguashan mine is a mine in China, located in Tongling city, Anhui province. Scholars are welcome to conduct academic research in mines. In this paper, only rock samples are taken from the mine, and there is no interest conflict. Test sampling has been approved by Dongguashan copper mine, therefore, no special permission is required for this research activity". To ensure homogeneity, a compact-structure cylindrical core with no obvious micro-cracks should be selected in the first place. According to the test requirements, the samples were made with two cylindrical dimensions of 50 mm×50 mm or 50 mm×100 mm, i.e., a height-diameter ratio of 1:1 or 2:1. In the meantime, all test specimens were polished carefully at both ends to make sure that the no parallelism and the no perpendicularity were both less than 0.02 mm. The rock samples with a height-diameter ratio of 2:1 are used for the triaxial compression test, while the rock samples with a height-diameter ratio of 1:1 are used for the three-dimensional high static load and frequent dynamic disturbance test.

### Test device

**Fig 1:**
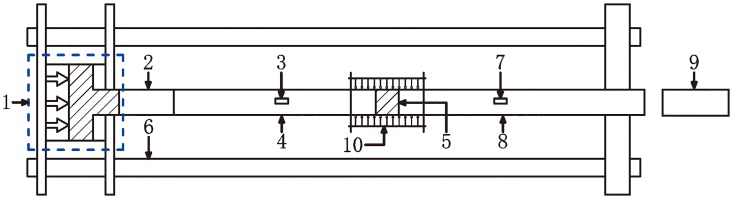
Structure diagram of the test load: 1-Pressure loading unit; 2—Buffer bar; 3—Strain gauge A2; 4—Transmission bar; 5—Rock specimen; 6—Support; 7—Strain gauge A1; 8—Incident bar; 9—Heterotype impact hammer; 10—Confining pressure device.

**Fig 2:**
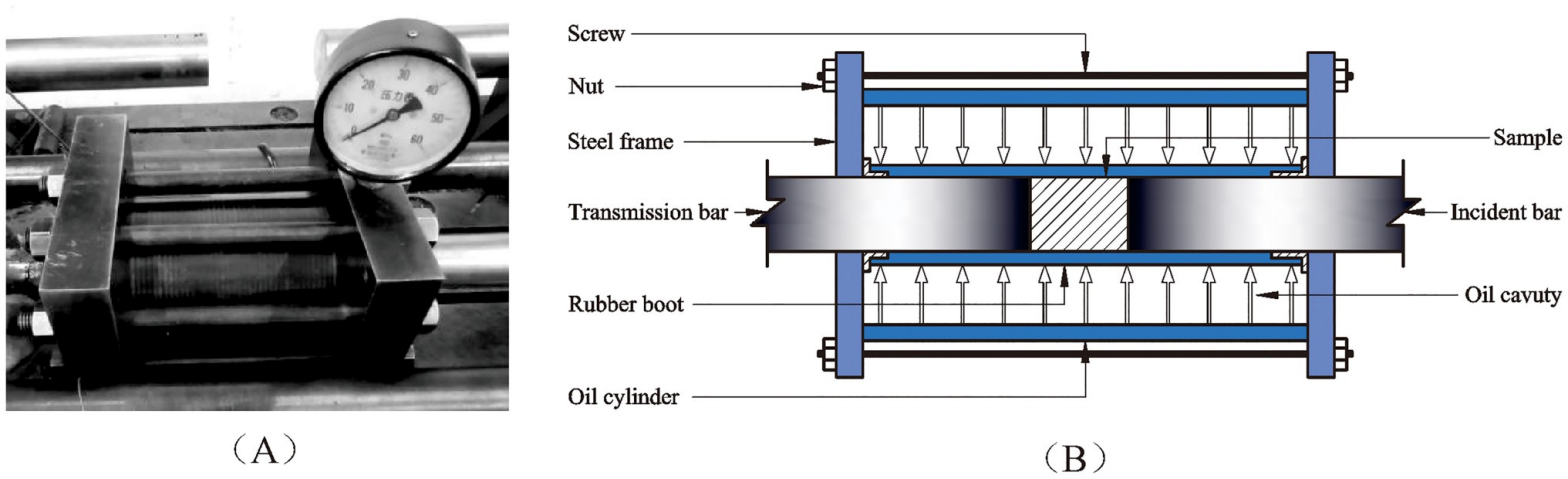
Physical picture and structural diagram of the confining pressure device. (A) Real picture of the confining pressure device; (B) Structural diagram of the confining pressure device.

The loaded structural representation of the improved SHPB test system is shown as Fig 1, which is used to investigate the dynamic mechanical characteristics and failure mode of serpentine under three-dimensional high static load and frequent dynamic disturbance [34–36]. The comparatively complicated loading of confining pressure affects the smooth progress of the experiment directly, so the real picture and structural diagram with the loading device for the confining pressure are shown in Fig 2. The diameter of the specimen is the same as that of the input bar and output bar, and the loading wave is a half-sine shock wave under a constant strain rate [37]. The data of each test is collected by a DL-750 oscilloscope and CS-1D ultra dynamic strain gauge.

### Test scheme

**Fig 3:**
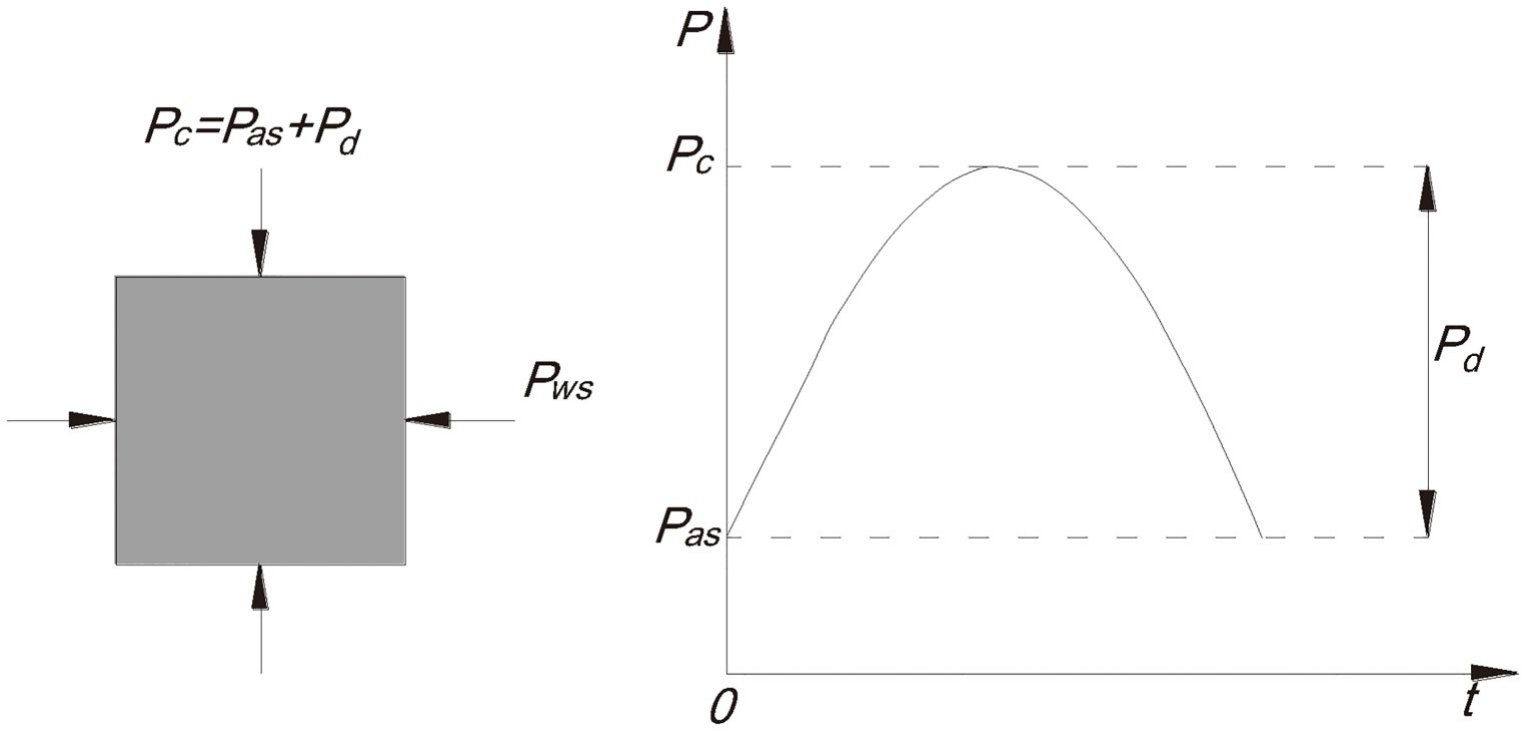
Sketch map of the mechanical model under the three-dimensional loaded test. *P*_c_—Total load; *P*_as_—Static load; *P*_d_—Impact load; *P*_WS_—Confining pressure.

The main content of the experiment is to pile a certain confining pressure upon the specimen beforehand and then impose higher axial static load. After stabilization of the foregoing load, the axial impact load of definite strength is applied to the specimen frequently until the specimen exhibits macroscopic destruction. It is necessary to keep the values of the confining pressure and axial compression constant throughout the entire test, and the test loaded mechanical model is shown in Fig 3.

To determine the values of the pre-loaded confining pressure and axial compression, the British Instron 1346 type electro-hydraulic servo testing machine was utilized to conduct the triaxial compressive strength test of serpentine. The test specimen was made into a cylinder with a diameter of 50 mm and a height of 100 mm. The test results are shown in Table 1.

**Table 1:** Test results of deep serpentinite under triaxial constringent experiments.

Specimen number	Confining pressure /MPa	Elasticity modulus /GPa	Triaxial compressive strength /MPa	Peak strain /10^−3^
SW1-1	5	14.34	142.87	13.44
SW1-2	10	19.27	171.90	15.66
SW1-3	15	14.85	185.36	17.19
SW1-4	20	12.98	208.04	19.43
SW1-5	25	14.81	225.76	20.54
SW1-6	30	11.36	249.02	22.89

Table 1 shows different triaxial compressive strengths of samples because of different confining pressures and internal structures. The pre-loaded confining pressure and axial compression in the three-dimensional high static load and frequent dynamic disturbance test are requested to approach peak strength under the condition of static load. At the same time, it is also important to ensure the nonoccurrence of macroscopic destruction. According to the compressive strength of deep serpentine under different confining pressures, a confining pressure value of 15 MPa is the referential standard. In this test, the axial compression is set to 100 MPa, 120 MPa, 140 MPa, and 160 MPa, and the confining pressure is set to 15 MPa, 20 MPa, 25 MPa, and 30 MPa. In addition, an impact air pressure value of 0.5 MPa is used to simulate the condition of small disturbance.

## Test results

### Characteristics of dynamic deformation

**Fig 4:**
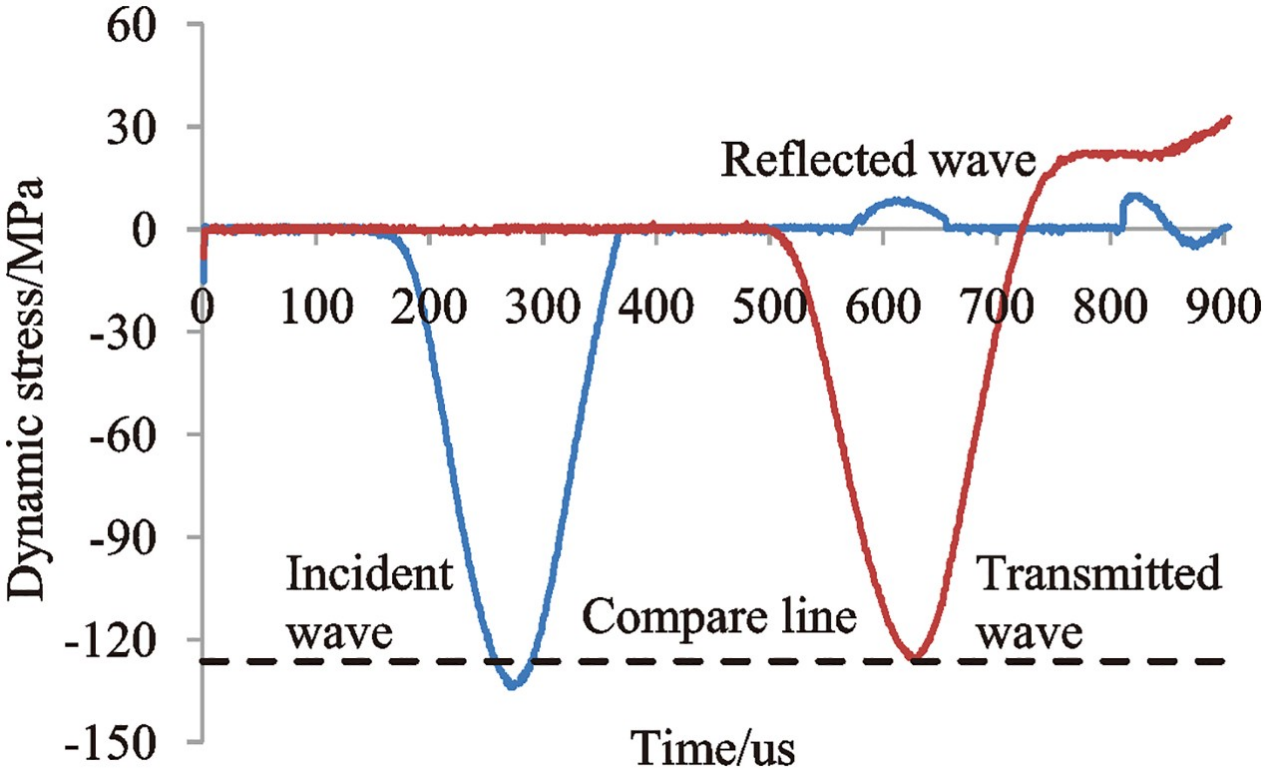
Stress-time curve of the impact test without a rock sample.

Before the impact test, the rock sample is not installed between the incident bar and the transmission bar, and the first impact occurs when the incident bar is in contact with the transmission bar at the same cross-section. At this moment, the stress equilibrium between the incident, transmitted, and reflected waves can be verified by the stress wave signal. Fig 4 shows the stress-time curve when no rock sample is installed. The amplitude of the incident wave is basically equal to the amplitude of the transmitted wave in Fig 4, which indicates that the stress wave is balanced during the impact test.

**Fig 5:**
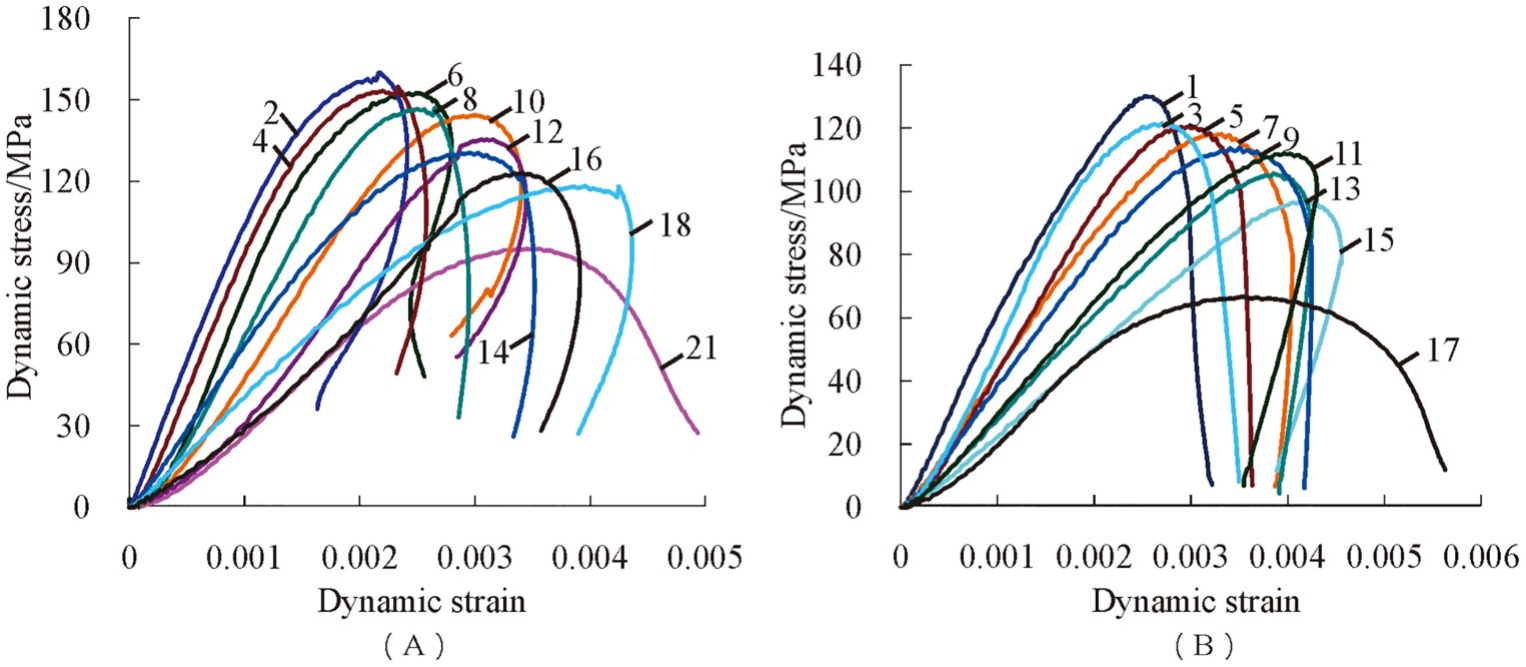
Dynamic stress-strain curves of serpentinite. (A) *P*_*ws*_ = 25 MPa, *P*_*as*_ = 120 MPa; (B) *P*_*ws*_ = 30 MPa, *P*_*as*_ = 160 MPa.

Due to the differences of confining pressure and axial compression, the test is divided into 16 groups. Dynamic stress-strain curves can reflect the features of the dynamic deformation of rock, so only two groups of typical dynamic stress-strain curves are listed in Fig 5 to save space. Only approximately evenly spaced stress-strain curves under impact disturbance are drawn in Fig 5 because of major impact instances. The number *n* in the previous diagram represents the number of impact disturbances.

As shown in Fig 5, with the increase of compression strain, the dynamic stress increases before the dynamic peak stress. In addition, there is no concave phenomenon at the initial stage of the curve during the beginning of several impacts. This is because the micro-cracks in the specimen have been closed under three-dimensional high static load. The compressive stress decreases gradually after the dynamic peak stress, while compressive strain shows an increasing trend on the whole. However, there also exists the phenomenon of partial rebound, as well as rebound strain. Because the next stage of dynamic peak stress is the unloaded stage, the compressive stress decreases naturally, but compressive strain increases with the combined action of axial high static and impact stress. Under the premise that the rock has not yet been destroyed macroscopically, compressive strain stops increasing when the impact stress is unloaded to zero. Otherwise, compressive strain rises sequentially under high static load until the sample is completely broken. The main reason for the generation of rebound strain is that the initial stored elastic force is greater than the impact stress, and the freed elastic energy resists the increase of compressive strain, which leads to diminution of compressive strain in small increments.

**Fig 6:**
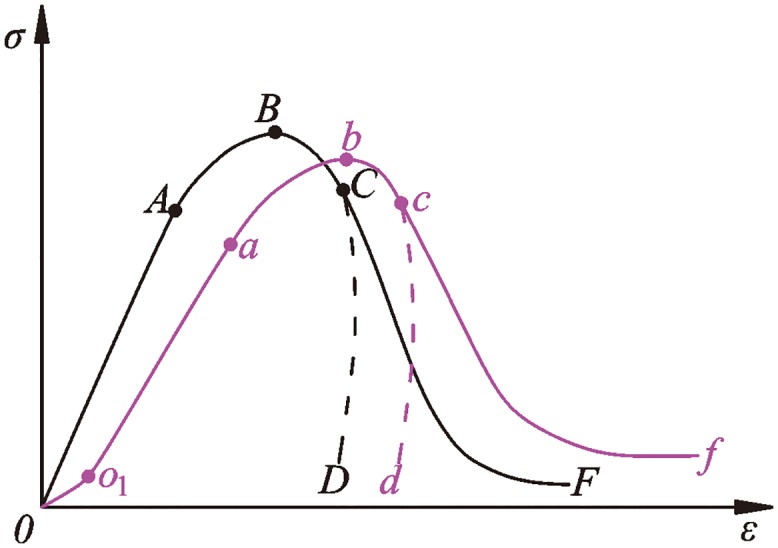
General characteristics of serpentinite on the dynamic stress-strain curve.

The general characteristics of the dynamic stress-strain curve of serpentinite subjected to frequent impact disturbance under three-dimensional high static load are further analyzed, and this can be divided into an elastic-plastic curve and plastic-elastic-plastic curve according to the curve form, as shown in Fig 6.

According to the morphological changes of two types of dynamic stress-strain curves, elastic-plastic curves include four stages: *OA*, *AB*, *BC* and *C*; plastic-elastic-plastic curves include five stages: *OO*_1_, *O*_1_a, *ab*, *bc* and *c*. Each stage shows that the rock samples have undergone different deformation characteristics.

Compaction stage (*OO*_1_ stage): This section of the dynamic stress-strain curve is convex, and the strain increment decreases with the increase of stress. When the preloading axial pressure is small or the rock sample is about to undergo macroscopic failure, the *OO*_1_ stage appears under impact load. One reason is that the small axial pressure of preloading cannot completely close the micro-cracks in the specimen. Another reason is that a large number of new cracks in serpentinite are generated before the macroscopic failure. In the initial stage of impact load, new cracks are compacted, and this causes the dynamic stress-strain curve to be convex.

Micro-crack steady development stage (*OA stage* or *O*_1_*a* stage): This section of the dynamic stress-strain curve develops in an approximately straight line, and the corresponding deformation modulus is the largest. This suggests that the rock at this stage is in the elastic deformation stage. When the micro-cracks in the rock are constant or exhibit steady development, the rock can be approximately considered to have the greatest impact resistance and the greatest difficulty of deformation under the impact compressive stress at this stage.

Micro-crack unstable propagation stage (*AB stage* or *ab* stage): This section of the dynamic stress-strain curve exhibits nonlinear development, and this curve increase trend gradually becomes slow. At the same time, the deformation modulus decreases gradually. This suggests that the rock at this stage is in the plastic deformation stage. When the micro-cracks in the rock are unstable, they can be instantly extended and penetrated, and the macroscopic failure of rock is caused under certain conditions. However, a single impact cannot make the micro-cracks in the rock specimen break through instantaneously because the dynamic disturbance impact stress is small. A single impact only acts as a disturbance and causes a state of unstable development.

Fatigue damage stage (*BC stage* or *bc* stage): This section of the dynamic stress-strain curve shows a dramatic declining trend. This phenomenon indicates that the impact compressive stress is in the unloading stage, and the serpentine specimen is in a state of damage. Both elastic deformation and plastic deformation occur in rocks. Rock damage accumulates with the increase of the number of impact disturbances. When the stress unloading is completed, even if there is no macroscopic failure of the rock, the internal structure of the rock has also changed. It is believed that the rock’s impact resistance is reduced at a macro level.

Fatigue failure stage (*C later stage* or *c later* stage): The specimen is macroscopically damaged in this stage. When the rock is not damaged, it is considered to be in an incomplete failure state. According to the different failure degrees of rock, the stress-strain curve shows two types of situations.

(1) Stress-strain curve rebound stage (*CD stage* or *cd* stage): The phenomenon of stress-strain curve rebound is that the strain decreases as the stress decreases. The reason is that, when the pre-loaded axial pressure does not cause the rock sample to become completely broken, there is a certain amount of elastic energy still stored in the specimen with fewer disturbance impacts. The elastic force stored in the rock sample is greater than the disturbance impact stress when the disturbance impact stress is in the unloading stage. The rebound phenomenon of the dynamic stress-strain curve is due to the small amplitude rebound of specimen deformation and the small amplitude reduction of dynamic strain.

(2) Stress-strain curve no-rebound stage (*DF stage* or *df* stage): The stress-strain curve does not rebound when the rock sample is completely broken under the disturbance impact load. The reason is that the elastic force stored in the rock sample begins to release before the maximum strain is reached during the impact unloading process, which causes the disturbed impact stress to be greater than the elastic force in the rock sample during the entire unloading stage. When the pre-added axial pressure is too high, macroscopic failure of the rock sample will occur. At this time, the role of disturbance impact is mainly to induce elastic energy release from the serpentinite specimen. The dynamic stress-strain curve does not rebound because the disturbance impact stress is greater than the stored elastic force in rock sample throughout the entire impact disturbance stage.

### Accumulative impact times of rock

**Fig 7:**
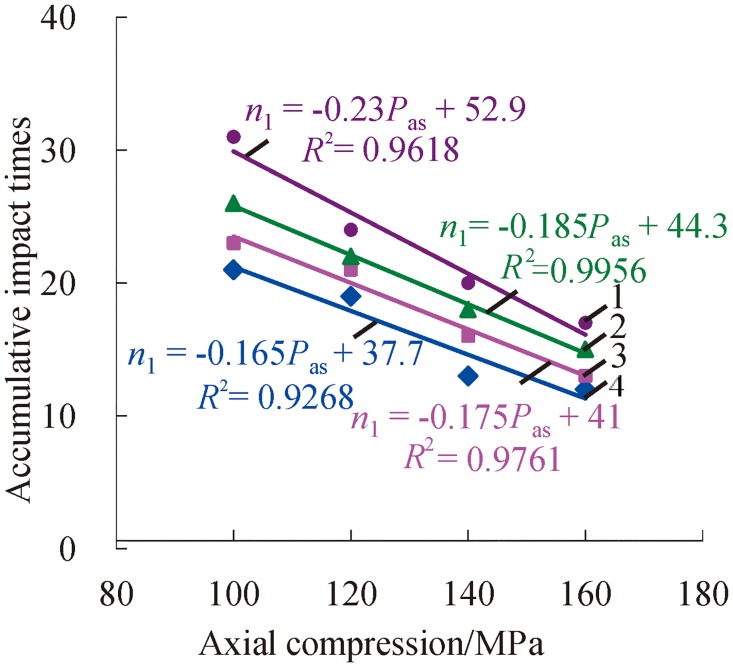
Change relations between accumulative impact times and axial compression with certain confining pressure (1—*P*_*ws*_ = 30 MPa; 2—*P*_*ws*_ = 25 MPa; 3—*P*_*ws*_ = 20 MPa; 4—*P*_*ws*_ = 15 MPa).

From Fig 7, it easy to see that accumulative impact times decrease with the increasing axial compression when the confining pressure is constant. The results prove that high axial compression promotes the expansion of a rock’s internal micro-cracks and the production of new micro-cracks, and it reduces a rock’s resistance to external shocks at the same time.

**Fig 8:**
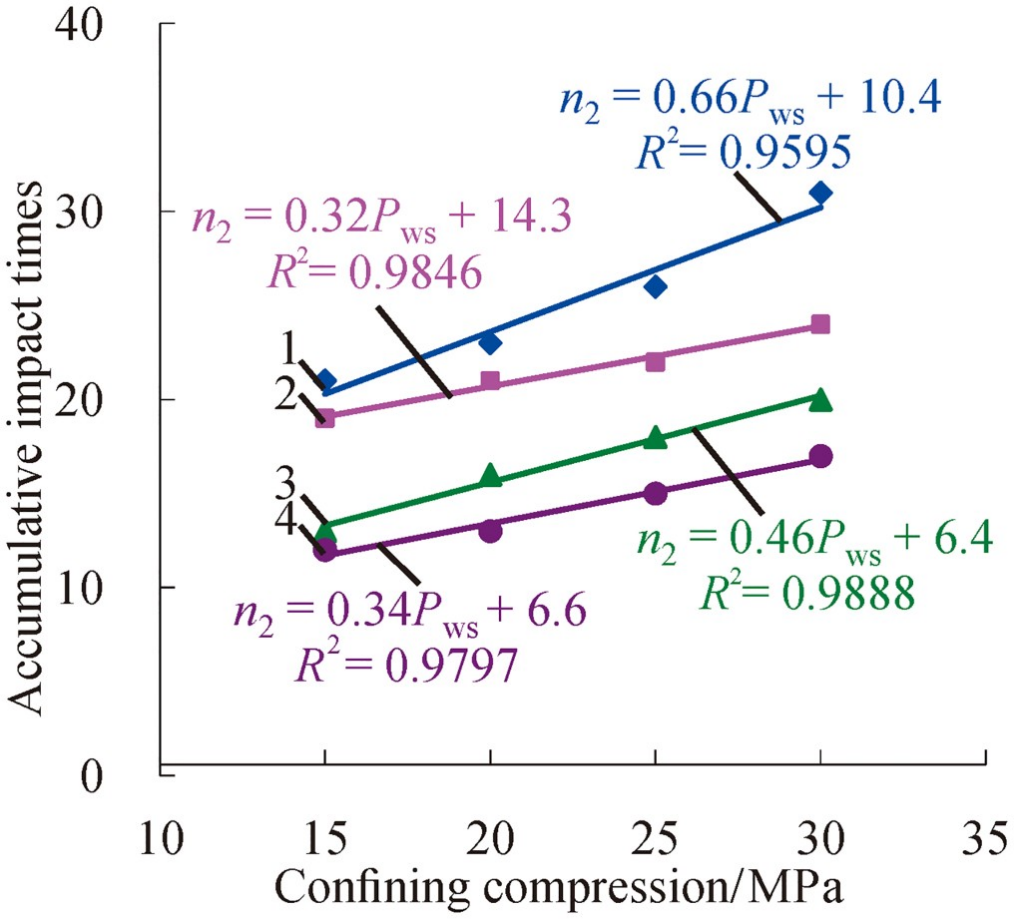
Change relations between accumulative impact times and confining compression with certain axial compression (1—*P*_*as*_ = 100 MPa; 2—*P*_*as*_ = 120 MPa; 3—*P*_*as*_ = 140 MPa; 4—*P*_*as*_ = 160 MPa).

Fig 8 shows that accumulative impact times increase with the increase of confining pressure when the axial compression is constant. The main reason for this is that confining pressure imposes restrictions on transverse germination, extension and connection of initial micro-cracks. In addition, confining pressure plays a role in the transverse compaction of samples. As a consequence, pre-loaded confining pressure enhances the resistant capacity of external shocks.

Based on a polynomial fit of the accumulative impact times and axial compression or confining pressure under three-dimensional high static load and frequent dynamic disturbance, the relation can be, respectively, presented as follows.

$$n_{1}=k_{1}P_{as}+C_{1}$$ (1)

$$n_{2}=k_{2}P_{ws}+C_{2}$$ (2)

where *n*_1_ and *n*_2_ are the accumulative impact times, respectively; *P*_*as*_ is the axial compression; *P*_*ws*_ is the confining pressure; *k*_1_ and *k*_2_ are the corresponding coefficients; and *C*_1_ and C_2_ are the corresponding constant terms.

In Figs 7 and 8, all of the values of fitting R ^2^ exceed 0.9, which present a nice fitting effect. According to analysis of the values of the coefficients (*k*_1_, *k*_2_), the results indicate that the coefficient *k*_1_ decreases with the increasing confining pressure. When the confining pressure exceeds a certain value, the reductive rate of accumulative impact times may be increased on the condition of increasing confining pressure. The coefficient *k*_1_ shows a decreasing trend in the mass with the increase of axial compression. That is to say, the increase rate of accumulative impact times may decrease due to the increasing axial compression.

**Fig 9:**
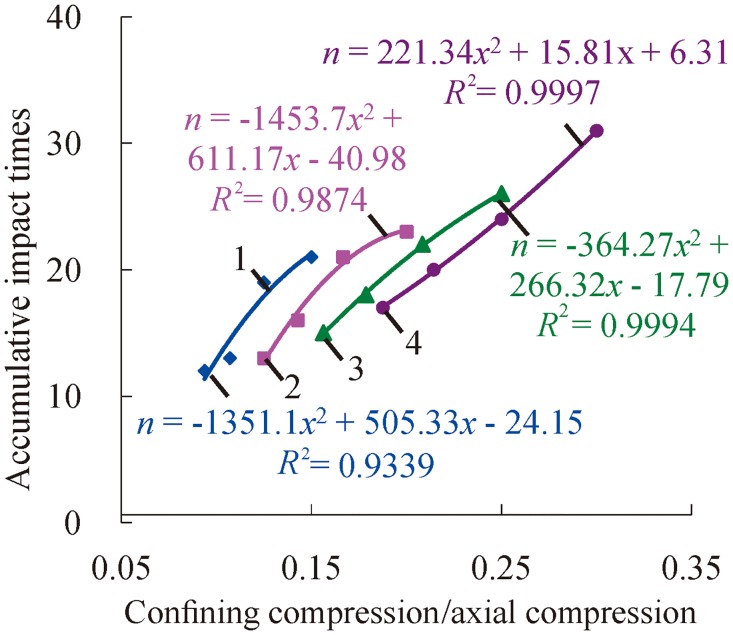
Change relations of accumulative impact times with the ratio of confining pressure to axial pressure (1—*P*_*ws*_ = 15 MPa; 2—*P*_*ws*_ = 20 MPa; 3—*P*_*ws*_ = 25 MPa; 4—*P*_*ws*_ = 30 MPa).

Based on the above analysis, the accumulative impact times were affected separately by confining pressure and axial pressure when the rock is under the condition of three-dimensional high static load and frequent impact disturbance. The accumulative impact times decrease as the axial pressure increases, and the accumulative impact times increase as the confining pressure increases. To analyze the influence of confining pressure and axial pressure on the accumulative impact times at the same time, the ratio of confining pressure to axial pressure was selected as a parameter variable to further analyze the change relations of the accumulative impact times, as shown in Fig 9.

From Fig 9, the relations of cumulative impact times with the ratio of confining pressure to axial pressure increases are quadratic polynomial. When the axial pressure is 15 MPa, 20 MPa and 25 MPa, there is an optimal ratio of confining pressure to axial pressure, and the specimens have the strongest resistance to external impact load when the ratio of confining pressure to axial pressure is optimal. When the axial pressure is 30 MPa, the accumulative impact times increase with the increase of the ratio, which is similar to the increase segment at the initial stage of low confining pressure. In general, the confining pressure within a certain range can enhance the rock’s ability to resist impact, but it also causes the damage of rock samples to intensify when the confining compression is too high. This phenomenon is similar to axial pressure. When the axial pressure is low, the rock can be compacted to improve its impact strength. When the axial pressure is too high, the rock will be damaged and destroyed. The change rule of the cumulative disturbance impact times with the ratio of confining pressure to axial pressure is consistent with the dynamic characteristic of deep rock, which is under three-dimensional stress states. The deep rock’s ability to resist impact is strongest when the three-dimensional stress is at the optimal ratio. At this time, it is not easy to damage when the deep rock is affected by drilling and blasting; otherwise, it is prone to rock burst and other disasters.

## Analysis of dynamic mechanical characteristic parameters

### Dynamic mechanical characteristic parameters

**Fig 10:**
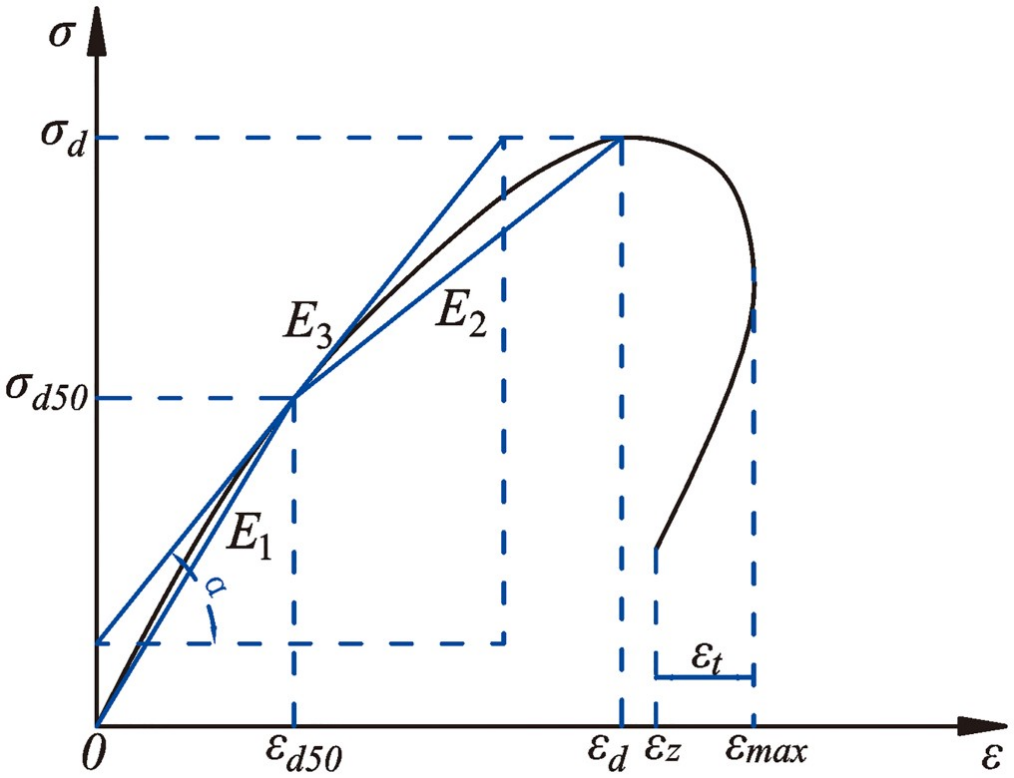
Picture of the dynamic mechanical parameters.

There is no definite stipulation regarding dynamic mechanical characteristic parameters at home or abroad. Therefore, for further study on the dynamic mechanical properties of rock, the typical dynamic stress-strain curves are selected to conduct analysis of the characteristic parameters. In this test, the defined schematic diagram of dynamic parameters of dynamic stress-strain curves after each impact is shown as Fig 10.

According to Fig 10, the computational formulas of deep rock’s dynamic deformation modulus and rebound strain after each impact disturbance are as follows.

$$E_{1}=\frac{\sigma_{\left(d50\right)}}{\varepsilon_{\left(d50\right)}}$$ (3)

$$E_{2}=\frac{\sigma_{\left(d\right)}-\sigma_{\left(d50\right)}}{\varepsilon_{\left(d\right)}-\varepsilon_{\left(d50\right)}}$$ (4)

$$E_{3}=\text{tan}\alpha$$ (5)

$$E_{d}=\frac{1}{3}(E_{1}+E_{2}+E_{3})$$ (6)

$$\varepsilon_{t}=\varepsilon_{\text{max}}-\varepsilon_{z}$$ (7)

where *E*_1_, *E*_2_, *E*_3_, and *E*_*d*_ are the secant modulus, second category of the secant modulus, deformation modulus of the loading section, and dynamic deformation modulus, respectively; *σ*_*d*_ is the peak stress, while *σ*_*d50*_ is half of the peak stress; *ε*_*d*_ is the peak strain, while *ε*_*d50*_ is the corresponding strain of half of the peak stress; *α* is the angle between the tangent of half of the peak stress and the *ε*-axis; and *ε*_*max*_, *ε*_*z*_, and *ε*_*t*_ are the maximum strain, final strain, and rebound strain, respectively.

### Change laws of the dynamic deformation modulus

**Fig 11:**
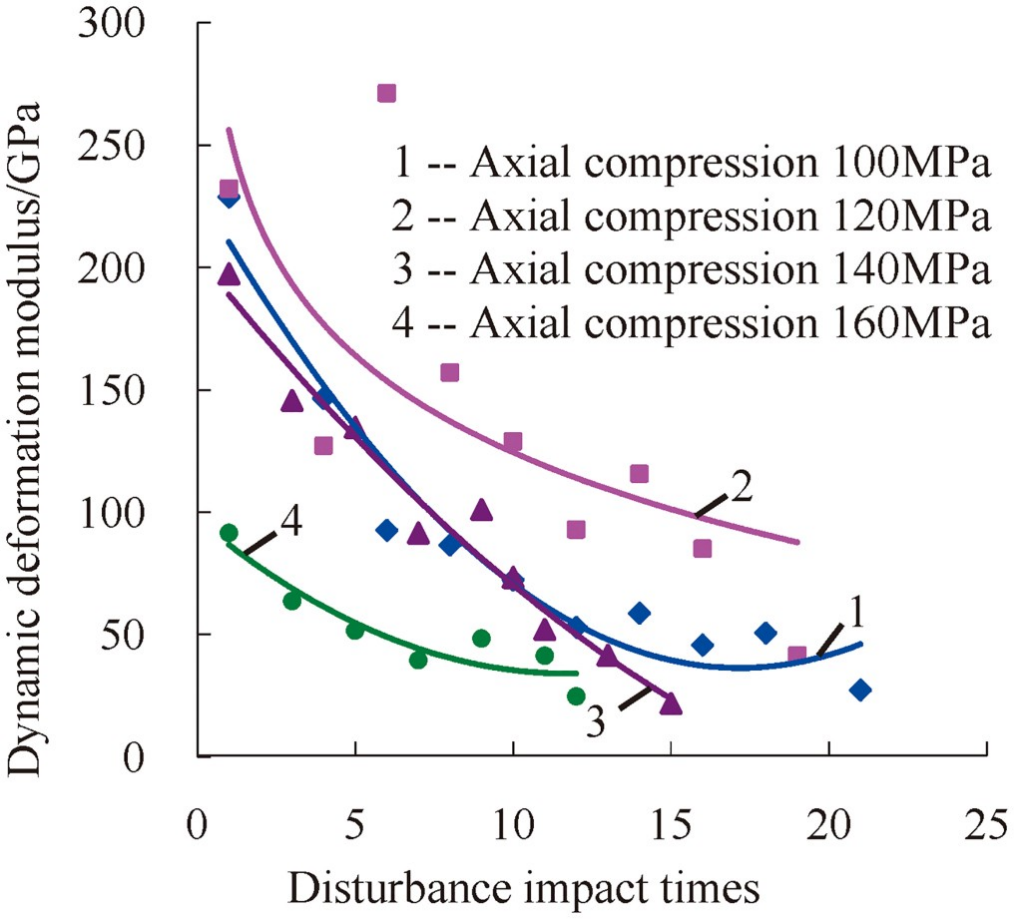
Change relations between the dynamic deformation modulus and accumulative impact times under a confining pressure of 20 MPa.

With the increase of impact times, the dynamic deformation modulus of serpentinite exhibits a gradually decreasing trend under three-dimensional high static load and frequent dynamic disturbance. Thus, the change law of the dynamic deformation modulus is as shown in Fig 11 when the confining pressure is 20 MPa.

Under the state of three-dimensional stress, although the confining pressure impedes a rock’s development of transverse deformation and enhances the resistant ability of rock to external impact load, internal micro-cracks of rock always go through a process of germination, extension, closure, and connection. All of these lead to perpetual accumulation of internal damage because of three-dimensional high static load and frequent dynamic disturbance. With the increase of impact times, the resistant ability of rock to external impact load decreases under the same stress status, i.e., the dynamic deformation modulus of rock exhibits a reductive trend.

### Change laws of the dynamic peak stress

**Fig 12:**
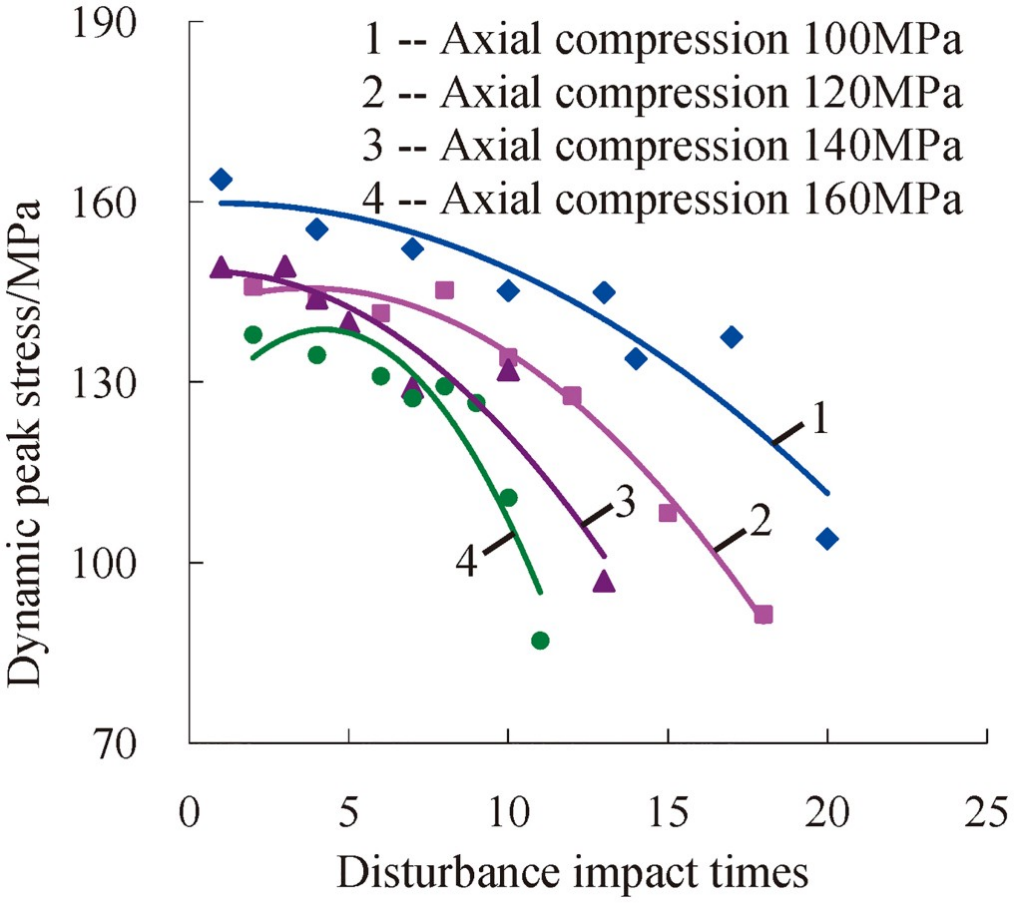
Change relations between the dynamic peak stress and accumulative impact times under a confining pressure of 15 MPa.

Fig 12 shows the change relations of dynamic peak stress under a confining pressure of 15 MPa. When the confining pressure and axial compression are certain, the dynamic peak stress of deep serpentine decreases with the increasing impact times under three-dimensional high static load and frequent dynamic disturbance. At this moment, the most important factor for resisting external shocks is impact disturbance times. There is a certain amount of damage to the rock after each shock under the condition of high static stress. Along with the accumulative damages, the ability of rock to resist shocks becomes weak gradually. In the end, rock loses resistance completely and exhibits macroscopic failure.

To research the influences of confining pressure and axial compression about resistant ability to impact disturbance, average values of dynamic peak stress are defined as the dynamic average strength of rock. The calculation formula of the defined "dynamic average strength of rock" is as follows:

$$\sigma_{\text{AS}}=\frac{1}{n}\sum_{i=1}^{n}\sigma_{\text{PS}i}$$ (8)

where *n* is a positive integer; *σ*_AS_ is the dynamic average strength of rock samples; *σ*_Psi_ is the dynamic peak stress that was obtained in the impact disturbance test, and the subscript i is the number of impacts.

**Fig 13:**
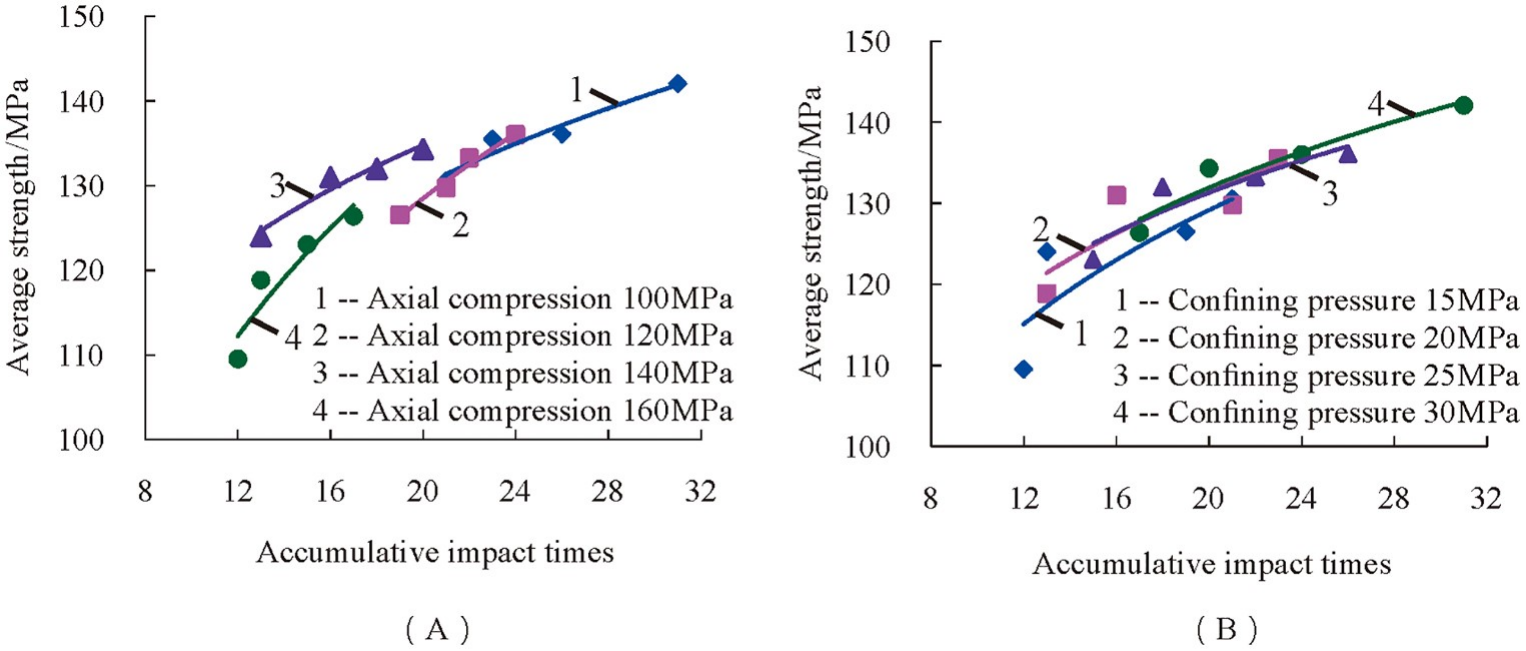
Relationship between the dynamic average strength of rock and the accumulative number of impacts. (A) The law of the dynamic average strength with the accumulative number of impacts when the preloading axial pressure is constant; (B) The law of the dynamic average strength with the accumulative number of impacts when the preloading confining pressure is constant.

To verify the rationality of the defined dynamic average strength, the law of its variation with the accumulative impact times is first analyzed. The dynamic average strength of rock samples was defined, and its variation rule with the number of impacts was analyzed, as shown in Fig 13. The figure shows that the dynamic average strength increases with the increase of the accumulative number of impacts. As shown in Fig 13A, the dynamic average strength increases with the increase of the accumulative number of impacts when the axial pressure of preloading remains constant. As shown in Fig 13B, the dynamic average strength also increases with the increase of the accumulative number of impacts when the confining pressure of preloading is constant. The more times the rock sample is subjected to disturbance impact, the stronger the rock can resist external impact. The law that the dynamic average strength increases with the increase of the accumulative number of impacts can also reflect the rock’s ability to resist impact disturbances. Through the above analysis, it is reasonable to define "dynamic average strength of rock" to describe the resistance of rocks to impact.

**Fig 14:**
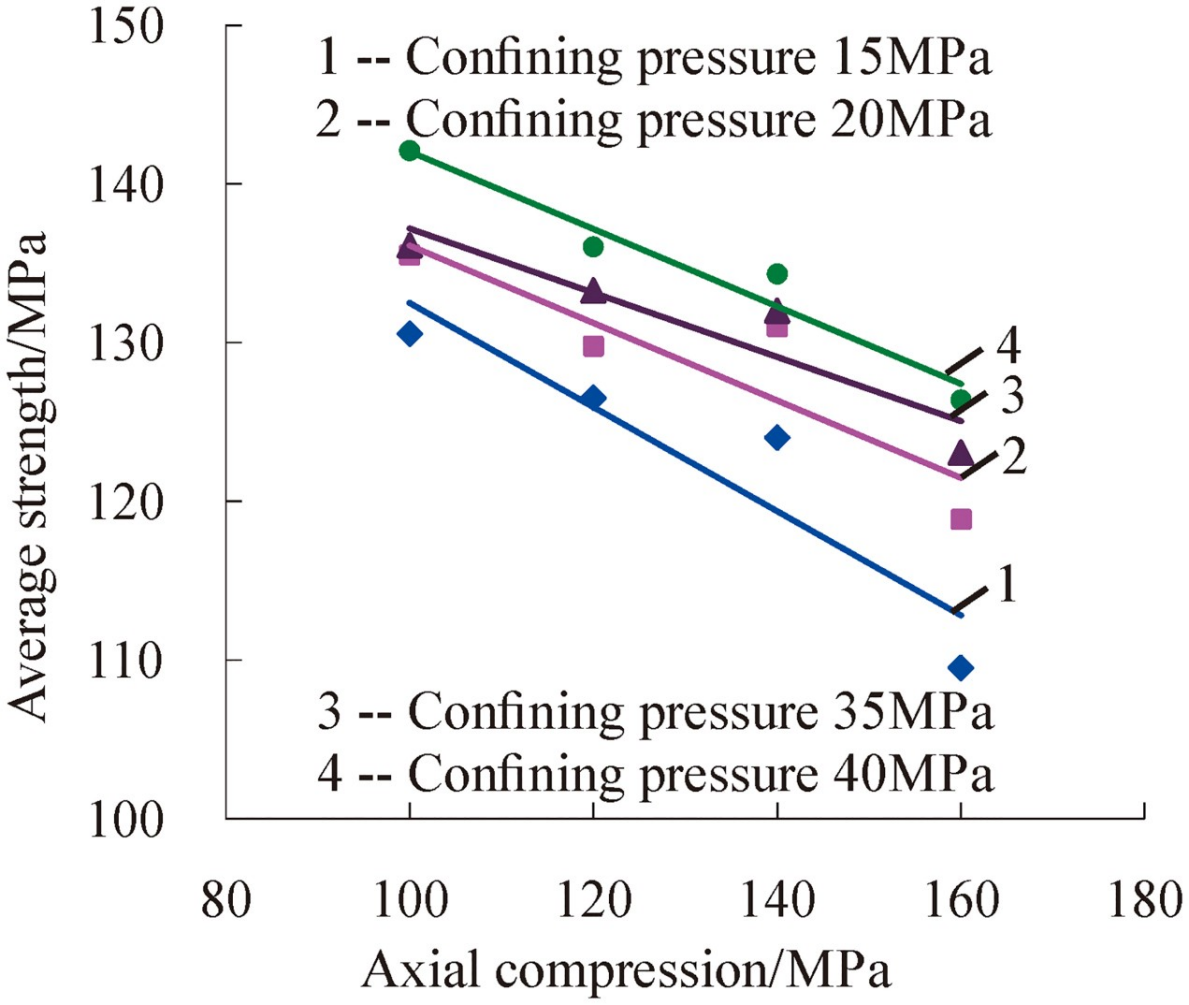
Change relations between dynamic average strength and axial compression.

Based on the above definitions, the relationship between the dynamic average strength and preloading axial and confining pressure was studied. It can be seen from Fig 14 that the dynamic average strength decreases with a linear trend due to the increasing axial compression. The main reason for this phenomenon is that rock’s internal micro-cracks extend and germinate continually under three-dimensional high static load and increasing axial compression. As a consequence, the ability of rock to resist external shocks decreases, as well as the accumulative impact times of same strength, namely, dynamic average strength decreases with the increasing axial compression.

**Fig 15:**
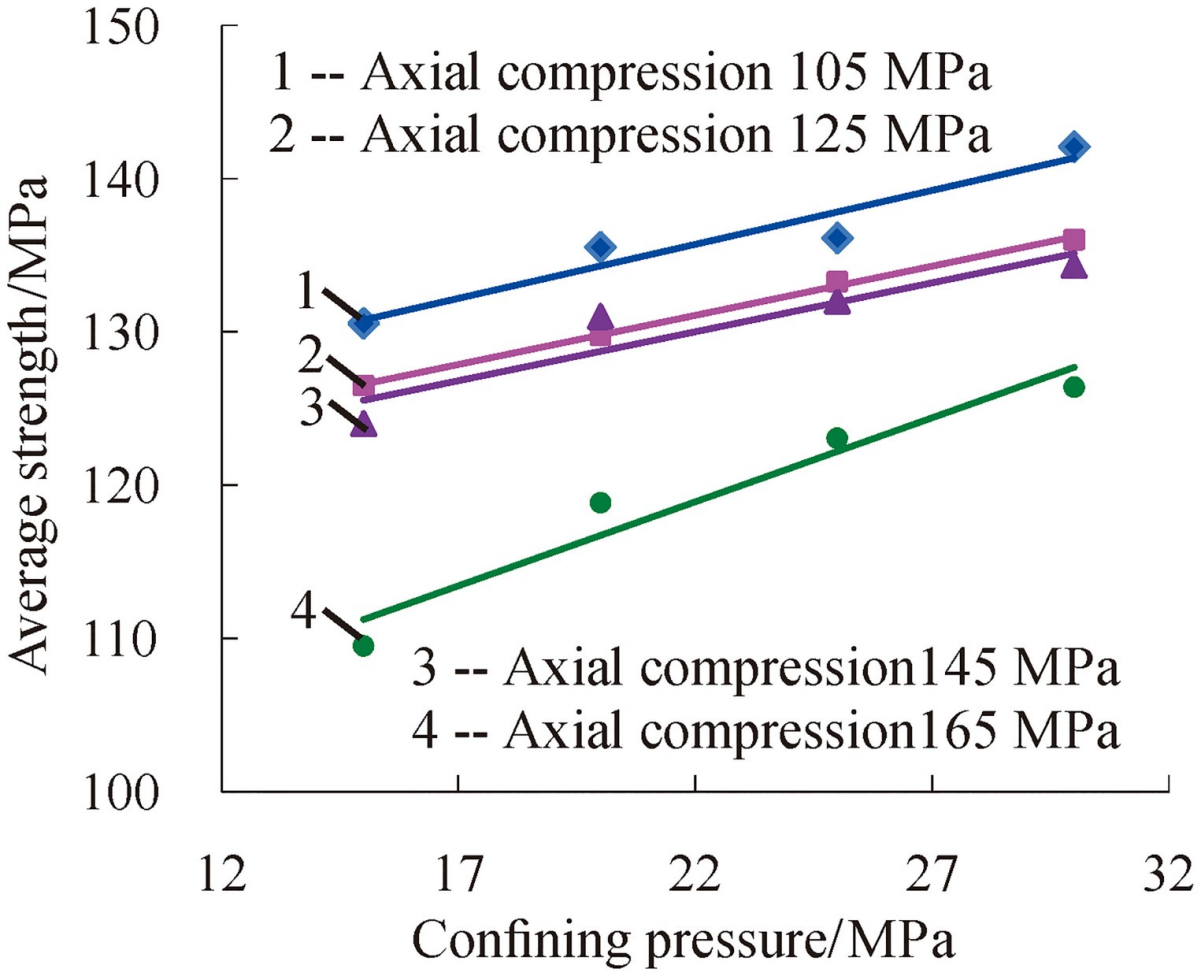
Change relations between the dynamic average strength and confining pressure.

Fig 15 shows that the dynamic average strength of serpentinite increases with a linear trend with increasing confining pressure. It is also proved that the effect of confining pressure on the damage degree evolution is affected by the grades of impulsive force under the same shock times. The transverse cracks in rock were limit development, and the germination and development of new micro-cracks were suppressed because the serpentinite specimen was under confining pressure. It is difficult for micro-cracks in rock to germinate and spread when the confining pressure is larger at a certain range. Therefore, the resistance of rock to external impact load is greater, which is reflected by the fact that the dynamic average strength increases with the increase of the confining pressure.

**Fig 16:**
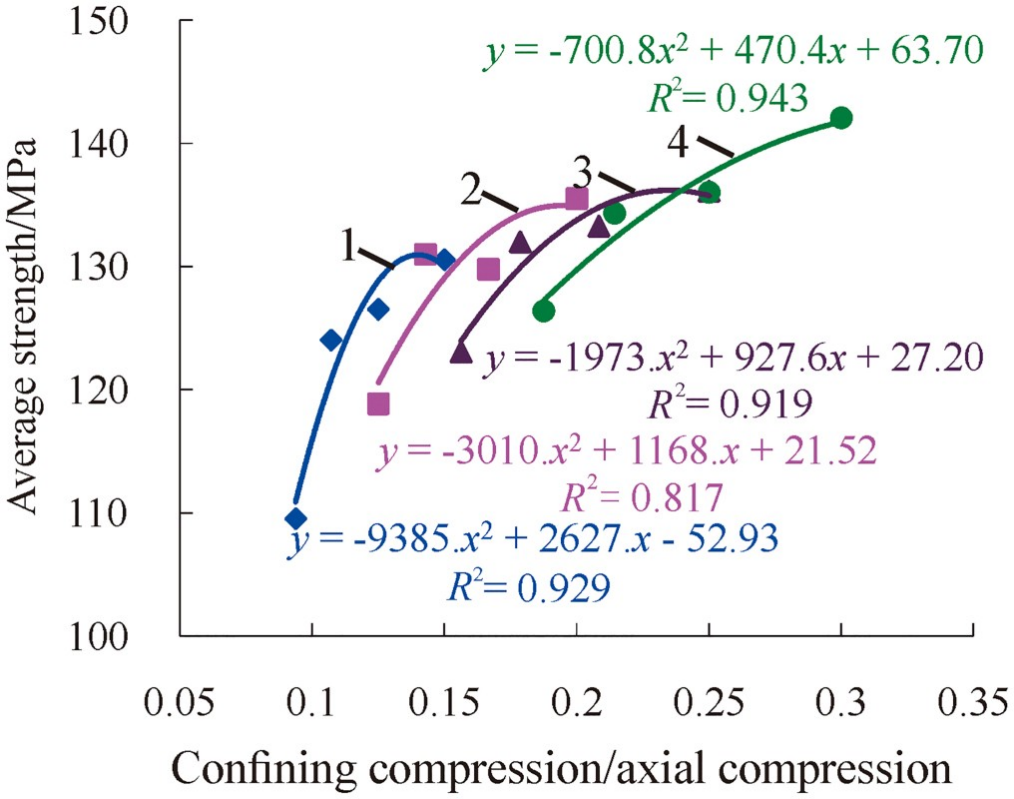
Change relations of dynamic average strength with the ratio of confining pressure to axial pressure (1—*P*_*ws*_ = 15 MPa; 2—*P*_*ws*_ = 20 MPa; 3—*P*_*ws*_ = 25 MPa; 4—*P*_*ws*_ = 30 MPa).

Based on the above analysis, the dynamic average strength of rock under three-dimensional high static load and frequent dynamic disturbance was affected separately by confining pressure and axial pressure. The dynamic average strength of rock decreases as the axial pressure increases, and the strength increases as the confining pressure increases. To analyze the influence of confining pressure and axial pressure on the dynamic average strength of rock at the same time, the ratio of confining pressure to axial pressure was selected as a variable parameter to further analyze the change relations of the dynamic average strength of rock, as shown in Fig 16.

Fig 16 shows that the change relations of the dynamic average strength of rock are similar to those of the accumulative number of impacts as the ratio of confining pressure to axial pressure increases, and their relationship was also quadratic polynomial. Based on this, it is considered that there is an optimal ratio of confining pressure to axial pressure. When the ratio of confining pressure to axial pressure is optimal, the dynamic average strength of specimens is maximized. The change relations of the dynamic average strength of rock with the ratio of confining pressure to axial pressure increases, once again proving that the deep rock’s ability to resist impact is strongest when the three-dimensional stress is at the optimal ratio. At this time, it is not easy to damage when the deep rock is affected by drilling and blasting; otherwise, it is prone to rock burst and other disasters.

### Change laws of maximum strain

**Fig 17:**
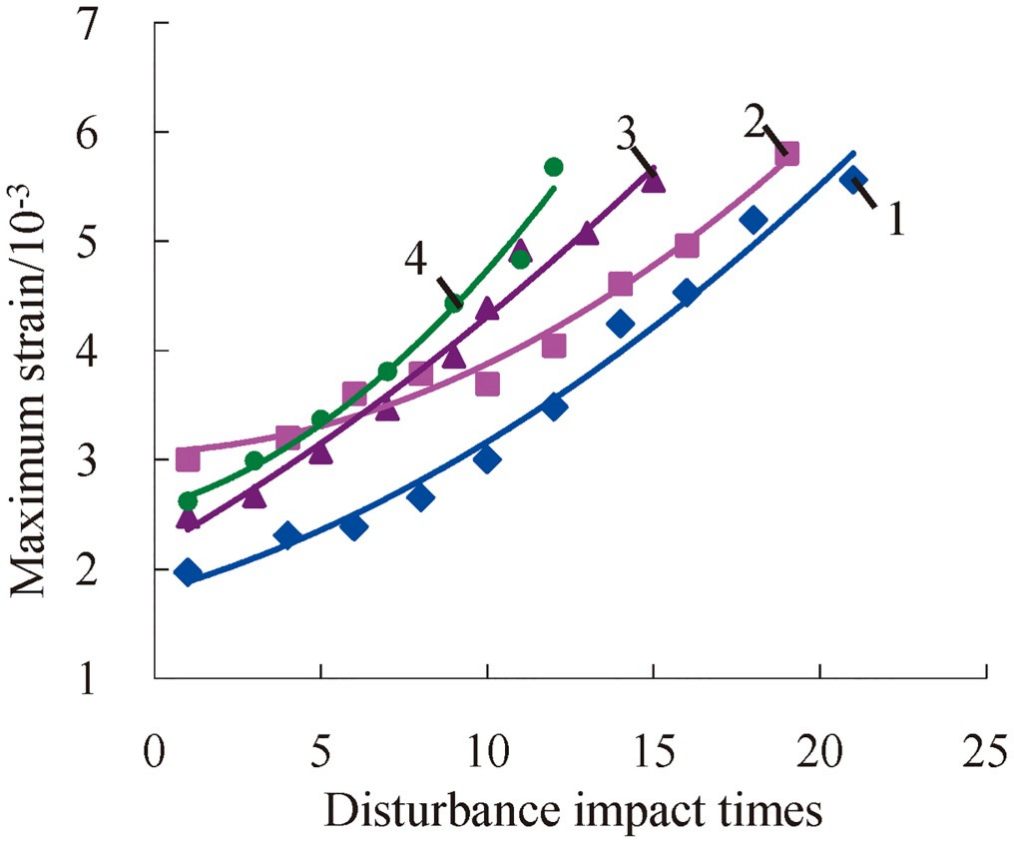
Change relations between the maximum strain and accumulative number of impacts under a confining pressure of 20 MPa (1—*P*_*as*_ = 100 MPa; 2—*P*_*as*_ = 120 MPa; 3—*P*_*as*_ = 140 MPa; 4—*P*_*as*_ = 160 MPa).

As shown in Fig 17, the maximum strain of deep serpentinite increases as the number of disturbance impacts increases when the deep serpentine is under three-dimensional high static load and frequent dynamic disturbance. The rock’s interior exhibits damages according to frequent impact disturbance, and the rock’s capacity to resist shocks decreases with the accumulative damages. In addition, samples are more likely to produce constringent deformation under the influence of the same impact disturbance, and both constringent strain and maximum strain increase relatively in the meantime. The metabolic rate of maximum strain is affected by the combined action of confining pressure and axial compression, so the incremental trend of maximum strain exhibits two different conditions, i.e., intersection or proximate parallelism. As shown in Fig 17, the change curve of the maximum strain of rock under 120 MPa of axial pressure intersects with the change curve of the maximum strain of rock under 120 MPa and 140 MPa axial pressure, while it is approximately parallel under the axial compression of 100 MPa, 140 MPa, and 160 MPa.

### Change laws of dynamic peak strain

**Fig 18:**
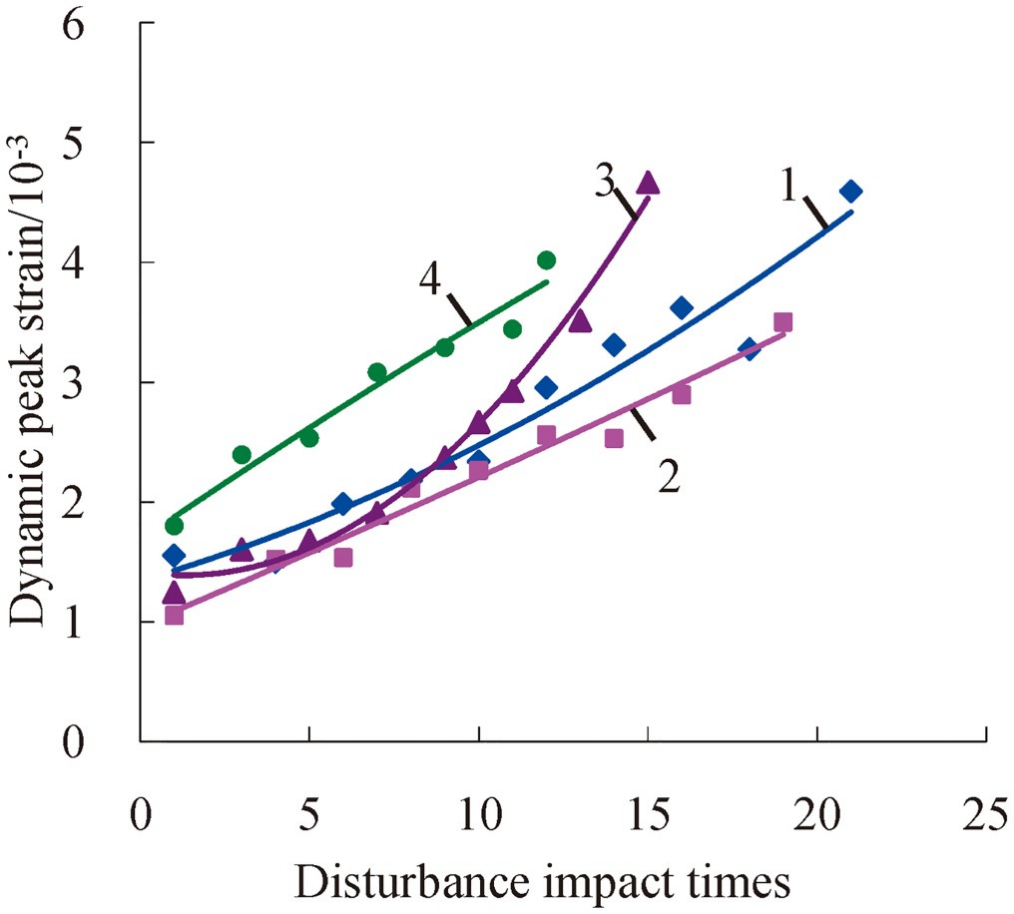
Change relations between the dynamic peak strain and accumulative number of impacts under a confining pressure of 20 MPa (1—*P*_*as*_ = 100 MPa; 2—*P*_*as*_ = 120 MPa; 3—*P*_*as*_ = 140 MPa; 4—*P*_*as*_ = 160 MPa).

With the increase of impact times, deep rock’s dynamic peak strain of the variational trend is similar to the trend of maximum strain, namely, both of them show an increasing trend. The trend line of dynamic peak strain also presents two different conditions from Fig 18, i.e., intersection and proximate parallelism, according to the contrasts of axial compression and confining pressure. As shown in Fig 18, the change curve of the dynamic peek strain of rock under 100 MPa of axial pressure intersects with the change curve of the dynamic peek strain of rock under 140 MPa of axial pressure, while it is approximately parallel under the axial compression of 100 MPa, 120 MPa, and 160 MPa. However, there still exist differences between them; the mutational condition of dynamic peak strain is more obvious than that of maximum strain under homologous axial compression and confining pressure. That is to say, the discreteness of the dynamic peak strain is greater than the maximum strain’s. Because rock sustains maximal impact force under the condition of dynamic peak strain, the existing micro flaws within the rock magnify flashily, and the mutational phenomenon of dynamic constringent deformation comes into being. After the dynamic peak strain, the rock’s status acquires a certain recovery in the stage of impact unload, and at the same time, the rock’s compressive strain trends towards approximately stable, and the mutational phenomenon becomes lesser.

### Change laws of resilient strain

**Fig 19:**
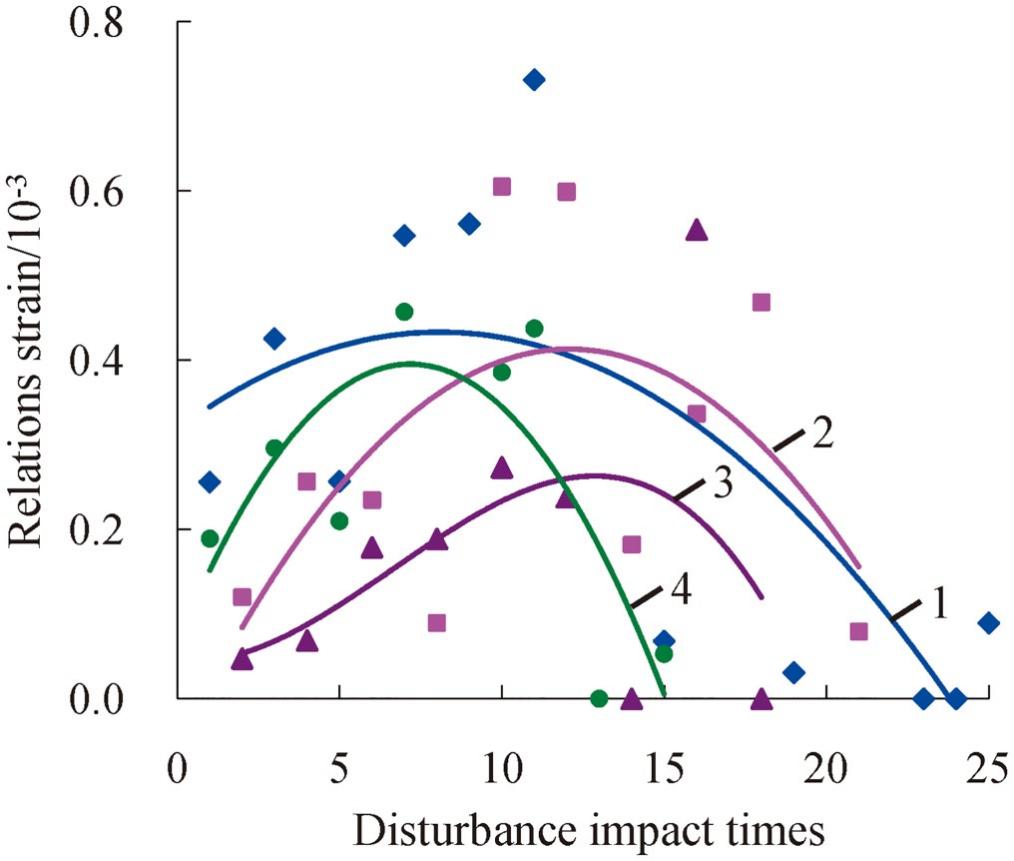
Change relations between the resilient strain and accumulative number of impacts under a confining pressure of 25 MPa (1—*P*_*as*_ = 100 MPa; 2—*P*_*as*_ = 120 MPa; 3—*P*_*as*_ = 140 MPa; 4—*P*_*as*_ = 160 MPa).

Rock exhibits the phenomenon of rebound at the later time of dynamic stress-strain curves in this test, which indicates that rock has elastic properties and elastic deformation under three-dimensional high static load and frequent dynamic disturbance. Fig 19 shows the change relations between the resilient strain and accumulative number of impacts when the axial pressures are 100 MPa, 120 MPa, 130 MPa, and 140 MPa and the confining pressure is 25 MPa.

Fig 19 shows that the resilient strain as a whole first increases and then decreases with increasing number of impacts. Moreover, the trend line of resilient strain is upwards convex, and resilient strain exhibits a value of zero. Meanwhile the discreteness of the resilient strain is comparatively large in each impact, namely, the mutational phenomenon of resilient strain is relatively serious. Generally speaking, rock produces elastic deformation under three-dimensional high static load and frequent dynamic disturbance, but transverse recovery of elastic deformation is prevented by confining pressure. As a result, restorable elastic deformation is random due to its non-dominated status, which is reflected in the mutational phenomenon of resilient strain. When the confining pressure and axial compression are constant, rock only produces infinitesimal damages or is not in the stage of initial impact disturbance. The elastic deformation of rock is small at this stage. However, elastic deformation increases gradually as the number of impacts grows, especially the restorable elastic deformation. This phenomenon eventually leads to an increase of the rebound strain. When the number of impacts reaches a certain value, rock’s ability to resist shocks is reduced on account of continually accumulated damages. In the meantime, elastic properties of rock weaken by degrees, as well as resilient strain. Finally, the macroscopic failure of rock occurs, while the stress-strain curves do not exhibit the resilient phenomenon.

## Failure process and characteristics

### Study of the serpentine sample failure process

**Fig 20:**
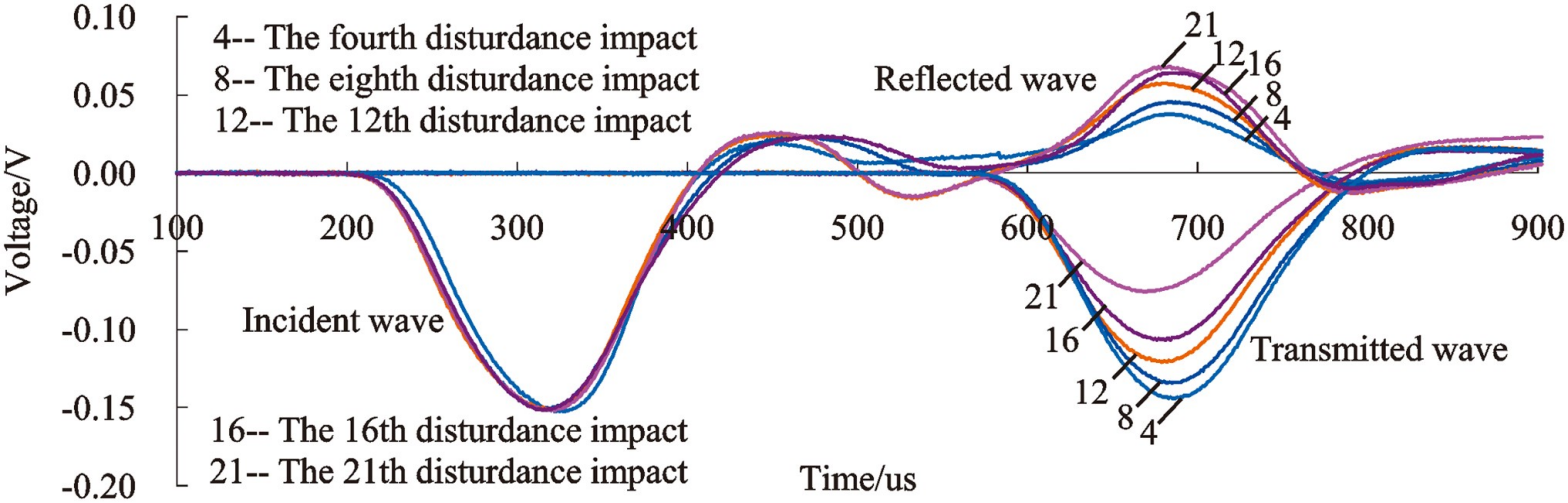
Typical time history curves of voltage under three-dimensional high static load and frequent dynamic disturbance (confining pressure of 20 MPa, axial compression of 120 MPa).

In the frequent impact disturbance test, the failure features of the specimens in the confining pressure device failed to be observed directly during the impact disturbance process. However, the failure features of the specimens can be identified indirectly by the change trends of the incident, reflected and transmissible voltage time history curves, which can be detected in the process of impact disturbance and can reflect the dynamic mechanical characteristics of specimens, as shown in Fig 20.

Fig 20 shows that the amplitude of the incident waves is consistent. The reason is that the energy of the incident stress wave is approximately equal when the shaped bullet is located in the same position in the firing chamber and the impact pressure is set at 0.5 MPa. However, rock damage increases and the energy of the reflected wave and transmitted wave change as the number of disturbance impacts increases, which is reflected in the difference in stress amplitude. The damage degree of the serpentinite specimen can be obtained by analyzing the variation rules of the amplitude of the reflected wave and transmitted wave.

As shown in Fig 20, the amplitude of the transmitted wave decreases with the rising number of disturbance impacts, which reflects that the damage degree of rock has been aggravated; meanwhile, the account of internal micro-cracks and micro-fracture surfaces are augmented with the development of disturbance impact. The incident wave is transmitted into the specimen and tends to be balanced after repeated reflections on micro-fracture surfaces, which causes a decreasing transmitted wave to pass through the specimen, reflecting a diminution in the amplitude. However, with the increase of the number of disturbance impacts, the amplitude of the reflected wave exhibits an upward trend. The rock failed to eject after destruction because of confining pressure constraints; as a results, the area at the end face of the rock is added for reflection, which implies the increasing reflection of stress wave, as well as the reflected energy.

### The failure characteristics of rock

#### The ultimate failure mode of rock

**Fig 21:**
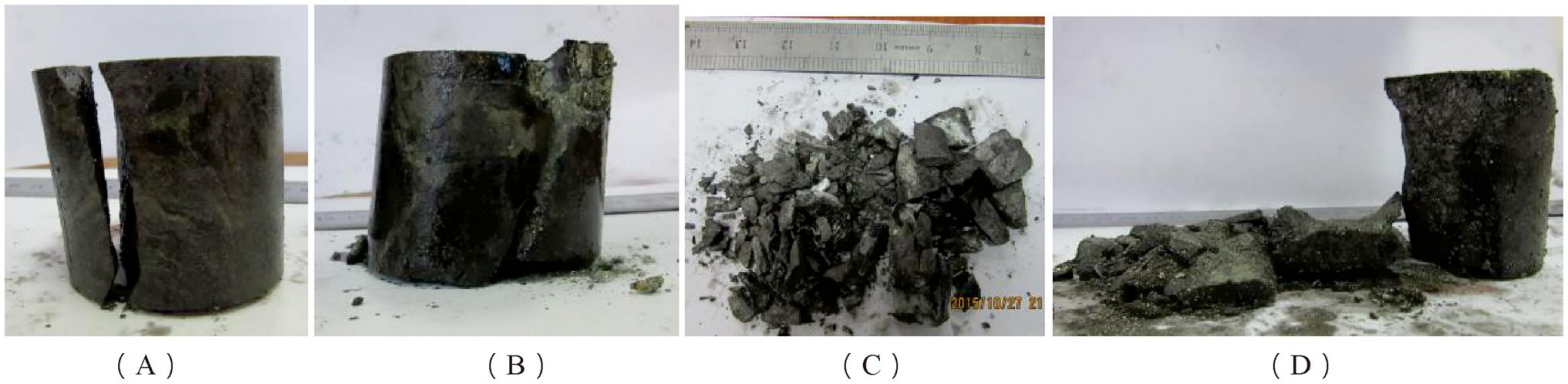
Typical failure mode of deep serpentinite under three-dimensional high static load and frequent dynamic disturbance. (A) Confining pressure of 25 MPa and axial compression of 140 MPa; (B) Confining pressure of 30 MPa and axial compression of 100 MPa; (C) Confining pressure of 15 MPa and axial compression of 160 MPa; (D) Confining pressure of 15 MPa and axial compression of 100 MPa.

The mechanical condition of rock and the interior defect of the rock structure can be reflected by the ultimate failure mode of rock. There are many factors influencing the failure characteristics of rock, and preloading axial pressure, confining pressure and frequent impact disturbance are the most important influencing factors. Those can be obtained by analyzing the influencing factors of the failure mode of rock under three-dimensional high static load and frequent dynamic disturbance. By analyzing the pictures of the damaged rock blocks after the test, it can be found that the tensile failure and the single-bevel plane shear failure are the main failure modes and that the pull-compression mixed friction failure is the auxiliary failure mode when testing the deep rock under three-dimensional high static load and frequent dynamic disturbance, as shown in Fig 21.

The broken rock appeared to be shaped like an arc or a long ribbon when the broken rock block was large. The shape can be determined by analyzing the shape of the broken rock block, as shown in Fig 21. This type of phenomenon indicates that part of the rock sample produced tensile failure. The fracture surface of the rock is smooth, and there is no obvious friction trace; with further observation, it is found that the rock samples were subjected to tensile failure. From the above, it can be considered that the failure mode of this part of the rock sample is tensile failure.

As shown in Fig 21B, the shapes of the broken rock blocks are mostly triangular pyramids, and the fracture surface intersects the axis of the rock sample, as well as interpenetrating the entire specimen when the broken rock block is small, which shows that the failure mode of rock is the single-bevel plane shear failure.

It was found that there is a layer of rock powder adhered to the fracture surface of the specimen, and there is more rock debris mixed in the broken rock blocks. This feature can be obtained by further analysis of the broken rock blocks of the serpentinite specimen, as shown in Fig 21C and 21D, which shows that the friction phenomenon is caused by the movement of one surface of the broken rock blocks against another in the serpentine sample failure process. At the same time, it can found that there are strip blocks and triangular pyramid blocks among the broken rock blocks, which indicates that the rock samples are subjected to both tensile and shear failure. Therefore, it can be considered that the failure mode of this part of the rock sample is the pull-compression mixed friction failure.

#### Influence of preloaded high axial pressure on rock failure characteristics

**Fig 22:**
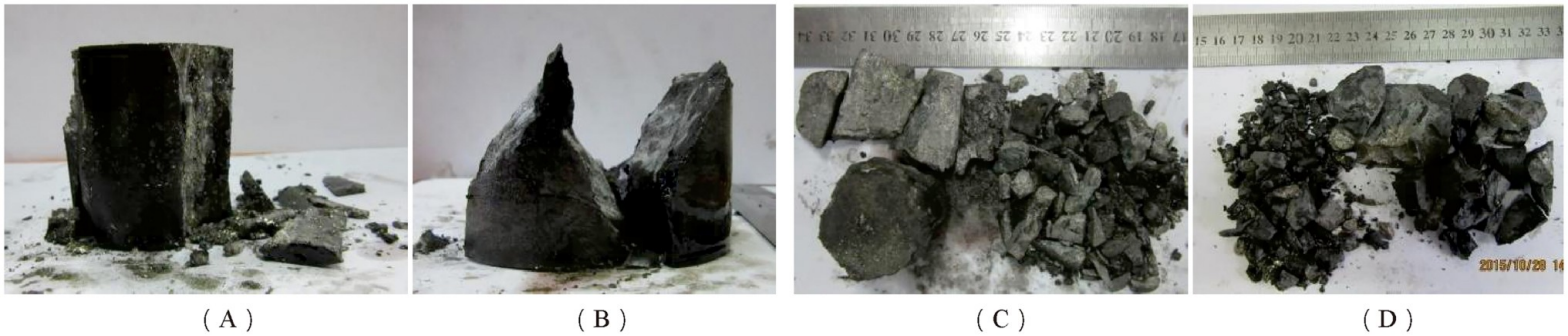
Failure mode of deep serpentinite under different preload axial pressures (confining pressure of 20 MPa). (A) Axial compression 100 MPa; (B) Axial compression 120 MPa; (C) Axial compression 140 MPa; (D) Axial compression 160 MPa.

In the three-dimensional high-static load frequent impact disturbance test, the main factors affecting rock failure characteristics are preloaded axial pressure and frequent impact disturbance. It was found that the broken rock block was smaller when the preloading axial pressure was larger by analyzing the ultimate failure lumpiness of the rock, as shown in Fig 22.

Fig 22 shows that the lumpiness of the broken rock block decreases obviously as the preloaded axial pressure increases from 100 MPa to 160 MPa when the confining pressure is maintained at 20 MPa. The rock samples are subject to the same transverse constraints when the preloading confining pressures are the same. At this time, the sample damage is more severe, and more elastic energy is stored in the rock when the preloading axial pressure is larger. New cracks in the rock are induced to germinate under three-dimensional high static load and frequent dynamic disturbance. The number of new cracks increases because the energy absorbed by micro-crack germination is sufficient when a large amount of energy is stored in the rock. There are more and more new cracks in the rock and the damage degree grows more and more severe with the increase of the number of impacts. Finally, macroscopic failure occurs in specimens along the penetrating micro-fissure surface, and the rock specimen is broken into many pieces. The result is that the failure lumpiness of the rock is smaller.

#### Influence of preloaded confining pressure on rock failure characteristics

**Fig 23:**
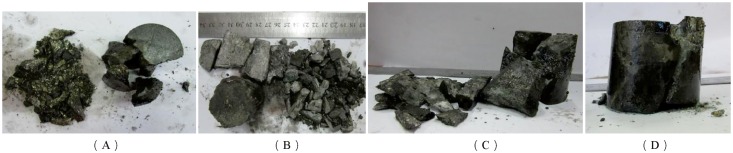
Failure mode of deep serpentinite under different preloaded confining pressures (axial compression of 140 MPa). (A) Confining pressure 15 MPa; (B) Confining pressure 20 MPa; (C) Confining pressure 25 MPa; (D) Confining pressure 30 MPa.

In the three-dimensional high-static load frequent impact disturbance test, the main factors affecting rock failure characteristics are the preloaded confining pressure and frequent impact disturbance. It was found that the broken rock block was larger when the preloading confining pressure was larger by analyzing the ultimate failure lumpiness of rock, as shown in Fig 23.

Fig 23 shows that the lumpiness of the broken rock block increases obviously as the preloaded confining pressure increases from 15 MPa to 30 MPa when the axial pressure is maintained at 140 MPa. The axial mechanical conditions of rock samples are consistent when the preloaded axial pressure is constant. The transverse micro-cracks in rock are restricted as the confining pressure increases, and the preloaded confining pressure enhances the capacity of rock to resist external shocks to a certain extent. The larger the confining pressure is, the more difficult it is for new cracks to germinate in the rock when the specimen is under frequent dynamic disturbance. The impact air pressure is only 0.5 MPa in the three-dimensional high-static load frequent impact disturbance test, and its function is to simulate small disturbance conditions. The impact disturbance is not enough to generate a variety of new cracks in the serpentine sample, but it only plays a role in that it induces the micro-crack to continue to expand and break through. At this time, there are two types of micro-cracks in the rock sample: one is germinated under preloaded axial pressure, and the other is the original micro-cracks in rock. Ultimately, the fracture surface is formed along the main rock crevice under frequent impact disturbance. Fewer micro-cracks are generated in the process of preloading confining pressure and axial pressure as the confining pressure increases when the axial compression is constant. Fewer fracture surfaces of the specimen growth under frequent impact disturbance, and this causes the amount of broken rock to be smaller and the failure lumpiness of the rock to be greater.

## Conclusions

With the Dongguashan Copper Mine as the engineering context, the test program was developed. The improved split Hopkinson pressure bar (SHPB) was used to study the dynamic mechanical characteristics and failure mode of deep serpentinite under three-dimensional high static load and frequent dynamic disturbance, and the main conclusions are as follows.

1. There are two types of dynamic stress-strain curves of deep rock under three-dimensional high static load and frequent dynamic disturbance: an elastic-plastic curve and plastic-elastic-plastic curve. The curve can be divided into five stages—the compaction stage, micro-crack steady development stage, micro-crack unstable propagation stage, fatigue damage stage, and fatigue failure stage—and the curves exhibit reductive phenomena of constringent strain after the dynamic peak stress because of the different degrees of rock damage. Moreover, these phenomena include two conditions, i.e., whether they rebound or not.
2. Both the dynamic average strength of rock and accumulative number of impacts decrease with the increase of preloaded axial compression and increase with the increase of preloaded confining pressure. When the ratio of confining pressure to axial pressure is optimal, the specimens have the strongest resistance to external impact load.
3. Both the dynamic deformation modulus and dynamic peak stress decrease with the increase of the accumulative number of impacts. However, the maximum strain and the dynamic peak strain increase with the increase of the accumulative number of impacts.
4. The phenomenon of rebound appears at the later time of dynamic stress-strain curves, and this is because the rock produces elastic deformation under impact load. The corresponding rebound strain as a whole first increases and then decreases with the increasing number of impacts.
5. For deep rock under three-dimensional high static load and frequent dynamic disturbance, tensile failure and single-bevel plane shear failure are the main failure modes, and pull-compression mixed friction failure is the auxiliary failure mode. The lumpiness of broken rock blocks decreases with the increase of preloaded axial compression and increases with the increase of preloaded confining pressure.
